# Mitogenome sequence accuracy using different elucidation methods

**DOI:** 10.1371/journal.pone.0179971

**Published:** 2017-06-29

**Authors:** Renata Velozo Timbó, Roberto Coiti Togawa, Marcos M. C. Costa, David A. Andow, Débora P. Paula

**Affiliations:** 1 Embrapa Genetic Resources and Biotechnology, Parque Estação Biológica, W5 Norte, Brasília, DF, Brazil; 2 University of Brasília, Campus Universitário Darcy Ribeiro, Brasília, Distrito Federal, Brazil; 3 Department of Entomology, University of Minnesota, 219 Hodson Hall, 1980 Folwell Ave., St. Paul, MN, United States of America; Xiamen University, CHINA

## Abstract

Mitogenome sequences are highly desired because they are used in several biological disciplines. Their elucidation has been facilitated through the development of massive parallel sequencing, accelerating their deposition in public databases. However, sequencing, assembly and annotation methods might induce variability in their quality, raising concerns about the accuracy of the sequences that have been deposited in public databases. In this work we show that different sequencing methods (number of species pooled in a library, insert size and platform) and assembly and annotation methods generated variable completeness and similarity of the resulting mitogenome sequences, using three species of predaceous ladybird beetles as models. The identity of the sequences varied considerably depending on the method used and ranged from 38.19 to 90.1% for *Cycloneda sanguinea*, 72.85 to 91.06% for *Harmonia axyridis* and 41.15 to 93.60% for *Hippodamia convergens*. Dissimilarities were frequently found in the non-coding A+T rich region, but were also common in coding regions, and were not associated with low coverage. Mitogenome completeness and sequence identity were affected by the sequencing and assembly/annotation methods, and high within-species variation was also found for other mitogenome depositions in GenBank. This indicates a need for methods to confirm sequence accuracy, and guidelines for verifying mitogenomes should be discussed and developed by the scientific community.

## Introduction

Mitochondrial DNA (mtDNA, mitochondrial genome or mitogenome) is used as a fundamental genetic marker in many research areas, including population genetics, evolutionary biology, phylogenetics and phylogeography, biodiversity studies, and molecular ecology [1–3]. Insect mitochondrial genomes are mostly about 15–22 kbp, circular, double-stranded DNAs that encode a set of 37 genes and a large control region (designated as the A+T-rich region in insects) [3,4]. The genes comprise 13 protein-coding genes (PCGs), 22 transfer RNAs (tRNAs) and two ribosomal RNAs (rRNAs). The A+T-rich region is responsible for regulating transcription and replication [5] and accounts for much of the mitogenome size variation across insect species [6].

Early elucidation of mitogenomes relied on long range PCR (LR-PCR) plus primer walking coupled with Sanger dideoxy sequencing. More recently, new approaches, using next-generation sequencing (NGS) have become common, including LR-PCR plus NGS, RNA sequencing (RNAseq) plus gap filling, and direct shotgun sequencing [3,7–9].

**Fig 1:**
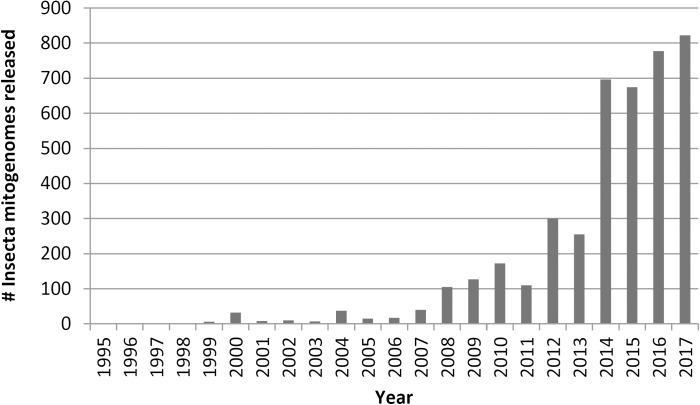
Release of Insecta mitogenome sequences in GenBank since 1995 (2017 covers only the first three months of the year). This information was obtained by searching *insecta[organism] AND mitochondrion[ti] AND genome[ti]* in the Nucleotide database and filtering the results by release date by using the "Release date" search criterion in the left navigation panel.

The assembly and annotation of the early mitogenomes usually relied on BLAST searches to identify protein-coding genes (PCGs) and covariance analyses to identify tRNAs [10], or the use of early assemblers (*e*.*g*., TIGR [11]; CAP [12]). Concomitant with the development of NGS, numerous methods for rapidly assembling (*e*.*g*., Celera [13]; SOAPdenovo, [14]; MITObim [15]) and annotating (*e*.*g*., DOGMA [16]; MOSAS [17]; MITOS [18]) mitogenomes have been developed and used [10, 19–21]. These exciting developments have caused the rate of mitogenome elucidation and deposition to increase tremendously (Fig 1). As a result, there are 4,213 Insecta mitogenomes deposited at GenBank (March 28^th^, 2017).

Public DNA databases (GenBank, DDBJ and EMBL) contain mitogenomes obtained through a variety of methodologies, which are often not specified by the depositor and lack an associated publication. Mitogenome sequences have originated from: libraries with pooled genetic material of several species [20,22] or with single species [23]; *de novo* assembly [20] or assembly using a previously sequenced reference genome or individual mito-genes from a target species as ‘seeds’ [15]; and annotation by tRNA identification (*e*.*g*., COVE) [24] followed by manual curation [20] or automated pipelines (MITOS) [18].

Despite acknowledgment that assembly and annotation software/methods vary considerably in their quality [25], there has been limited information about the accuracy of the deposited sequences and their annotations, introducing uncertainty about the mitogenome sequences. Public DNA databases are repositories (libraries) and the depositor is expected to provide sequences that meet basic quality criteria regarding the PCG annotations (*e*.*g*., presence of start and stop codons, no internal stop codon, etc). The depositor is assumed to have accurate sequences and is not required to provide methodological detail or a proof of quality.

Pooling multiple species in a single library would reduce the cost of library construction compared to single species libraries [20,22], but may increase the probability of interspecific chimeras, resulting in less accurate sequences. *De novo* assembly methods may generate more fragmented assemblies, but use of reference genomes may bias the sequence to the reference or propagate errors in the reference. In this work, we examined the effect of using different sequencing methods (number of species in a library, insert size, sequencing platform) and different assembly and annotation methods in the elucidation of mitogenomes of three species of ladybird beetles.

## Material and methods

### Sample preparation and DNA shotgun-sequencing

Adult *Cycloneda sanguinea*, *Harmonia axyridis* and *Hippodamia convergens* (Coleoptera: Coccinellidae) were collected from agricultural fields in the Federal District, Brazil. All collections were authorized by SISBIO (authorization number 36950), and access to the genetic heritage and transportation of biological material were authorized by IBAMA (authorization number 02001.008598/2012-42). These species are widespread and important natural enemies of agricultural pests in Brazil and other countries. Three specimens of each species (about 25 mg of body weight) were used to extract the total DNA separately from each species by DNAeasy Blood and Tissue Kit (Qiagen, Germany), following the manufacturer’s instructions. The purified DNA was quantified with Qubit HS (Invitrogen, USA). Two sequencing methods were used. In the first, the three species were pooled without individual tagging after the DNA in the samples was normalized to an equal concentration of 1 μg to make one TruSeq Nano library (Illumina, USA). The library was sequenced on the MiSeq (2×250 bp, insert size 850 bp, 500 cycle kit) using 15% of the flowcell (Illumina, USA). In the second, single species libraries were used with 1μg of DNA per sample to construct individual TruSeq Nano libraries and sequenced with HiSeq 2500 Rapid Mode (2×250 bp, insert size 550 bp, 250 cycle kit) using 2% of the flowcell (Illumina, USA). We switched to the HiSeq platform due to its much higher sequencing depth and cost-effectiveness.

### Mitogenome assembly

Two mitogenome assembly methods were used: *de novo* and using a reference mitogenome. The first method consisted of mining reads in the dataset with similarity to Insecta mitogenomes available at GenBank, using the BLASTn algorithm (BLAST v2.2.27; E-value≤1e-5; maximum target sequences 1; DUST filtering disabled [26]), according to Crampton et al. [20]. The mined mito-reads were assembled using Celera v7.0 (CA) [13] and IDBA-UD v1.1.1 [27]. For Celera, we used largely default settings without any additional pre-processing of the reads (doToggle = 1; toggleUnitigLength = 500; unitgger = bogart). Prior to assembly with IDBA-UD, the reads were passed through a quality control step with PrinSEQ-lite v0.19.2 [28] to trim low quality bases and remove short sequences (minimum length 150 bp; trim 3’ bases below Q20; minimum mean quality Q25; no Ns). Pairs where both reads passed quality control were extracted using a cdbfasta pipeline [20] and converted to Fasta format. The scaffolds generated by both assemblers were concatenated in Geneious v7.0.5 [29] using the parameters: no gaps allowed, minimum overlap 150, maximum mismatches per read 0, minimum overlap identity 99%, maximum ambiguity 1.

To assign the assembled mitogenomes to particular species in the pooled samples, we used barcodes *cox*1 and *cyt*b as ‘baits’. Fragments of the 5’ portion (‘barcode region’) of the mitochondrial genes *cytochrome oxidase 1* (*cox*1) and *cytochrome oxidase b* (*cyt*b) were determined independently for each species by sequencing the PCR amplicons generated respectively by the mitochondrial universal primer pairs Jerry & Stev-Pat_R and Sytb-F & Sytb_R, according to Timmermans et al. [30]. The amplicons were checked on 1% agarose gel, purified using QIAquick PCR Purification kit (Qiagen), and sequenced by Sanger methodology (ABI3700 sequencer). The barcode baits were assigned to the assembled mitogenomes using the Custom BLAST tool (E-value = 0) in Geneious v7.0.5 [29], selecting hits with a minimum length of 95% of the query coverage with at least 98% identity.

In the second method, the quality of the raw dataset was evaluated by FastQC v0.11.3 (http://www.bioinformatics.babraham.ac.uk/projects/fastqc/) and the adaptors were trimmed by Fastqc-mcf v1.04.807 [31] and Cutadapt v.1.9.1 [32] before assembly. The mitogenomes were assembled by MITObim v1.8 [15] using as reference the mitogenome of the coccinellid *Coccinella septempunctata* (GenBank: JQ321839.1) and the 5’ portion of the genes *cox1* and *cytb* of each target species as baits. The same reference mitogenome was used for the three target coccinellid species because it was the most closely related species to all three targets that was available at GenBank at the time of the analysis. The reference and all three targets are in the subfamily Coccinellinae and tribe Coccinellini, and no other species in the tribe was available at GenBank to use as a reference, except for *H*. *axyridis*. However, we considered that using *H*. *axyridis* as a reference would unduly bias the assembly of the mitogenomes.

The sequencing coverage [33] for each of the four elucidated mitogenomes for each species was obtained by mapping the reads (Q20<90%) in a source library to the respective mitogenome with Geneious v7.0.5 [29] using the parameters: no gaps allowed, minimum overlap 30, maximum mismatches per read 0, minimum overlap identity 99%, maximum ambiguity 4. For methods 2 and 4, in which the assembly method was MITObim, we mapped the reads retrieved in the final iteration.

### Annotations

Two annotation methods were used. The first was used with the *de novo* assembly method and consisted of annotating the obtained mitogenome scaffold for the expected tRNA sequences using COVE v2.4.4 [24] with covariance models of Coleoptera [30] according to Crampton et al. [20]. The annotated tRNAs were imported to Geneious v7.0.5 [29] and the target mitogenome was aligned with the annotated mitogenome of the coccinellid *Co*. *septempunctata* (GenBank: JQ321839.1), which was used again as a reference, using standard parameters in MAFFT v7.017 [34]. After this, manual curation was performed to check: a) the expected order of the PCGs and tRNAs; b) the match of the predicted open reading frames (invertebrate mitochondrial genetic code) with the pattern of the reference mitogenome (*cox1* does not start with an ATG as methionine and some *nad* genes start with ATT); c) the presence of stop codons in the middle of the coding regions of the target sequence; d) any deletions or insertions that would change the translation frame; e) the stop codons at the end of the coding sequence (the mitochondrial stop codons are TAA, TAG, TA or just T); and f) the orientation of the genes (*e*.*g*., some *nad* genes are reverse-oriented). Then, the annotation was transferred to the target mitogenome (selection “Annotation”>“Copy to all selected region”), and the annotated target mitogenome was extracted from the alignment. Mitogenome completeness was checked when the length was longer than 16 kb and when both *nad*2 and *12*S rRNA were present by searching for overlap between the starting and ending regions by aligning the first and last few 100 bp using MAFFT v7.017 [34].

For the second method, which was used with the MITObim assembly method, mitogenome annotations were carried out by MITOS (v763), available for online use at http://mitos.bioinf.uni-leipzig.de/ [18]. The sequences (Fasta format) of the mitogenomes were uploaded individually for each species to the MITOS server, using the default parameters and invertebrate genetic code. The GFF files generated by MITOS were imported to Geneious v7.0.5 and combined with the original Fasta sequences.

The annotated mitogenomes generated using the combination of all these methods (Table 1) were deposited at GenBank (KJ778883, KJ778886, KJ778888, KU877027, KU877028, KU877030, KX755335, KX755334, KX755332, KU877170, KX755330 and KX755331) using the Banklt submission tool.

**Table 1:** Methods used to obtain and annotate the coccinellid mitogenomes.

	Method 1	Method 2	Method 3	Method 4
Sequencing method	Pooled DNA samples, library insert size 850 bp, Illumina MiSeq platform	Pooled DNA samples, libarey insert size 850 bp, Illumina MiSeq platform	Single DNA sample, library insert size 550 bp, Illumina HiSeq platform	Single DNA sample, library insert size 550 bp, Illumina HiSeq platform
Assembly	*de novo*	MITObim	*de novo*	MITObim
Annotation	customized	MITOS	customized	MITOS

### Mitogenome comparisons

The mitogenomes from the four different methods (two sequencing methods each with two assembly/annotation methods) were compared regarding number of genes obtained and sequence identity using MAFFT alignment v7.017 [34] with the algorithm auto, scoring matrix BLOSUM62, gap opening penalty 1.53, and offset value of 0.123 within Geneious v7.0.5. Annotations were compared regarding gene location/position, length, and order, number of genes annotated and gene transcriptional direction.

## Results

**Fig 2:**
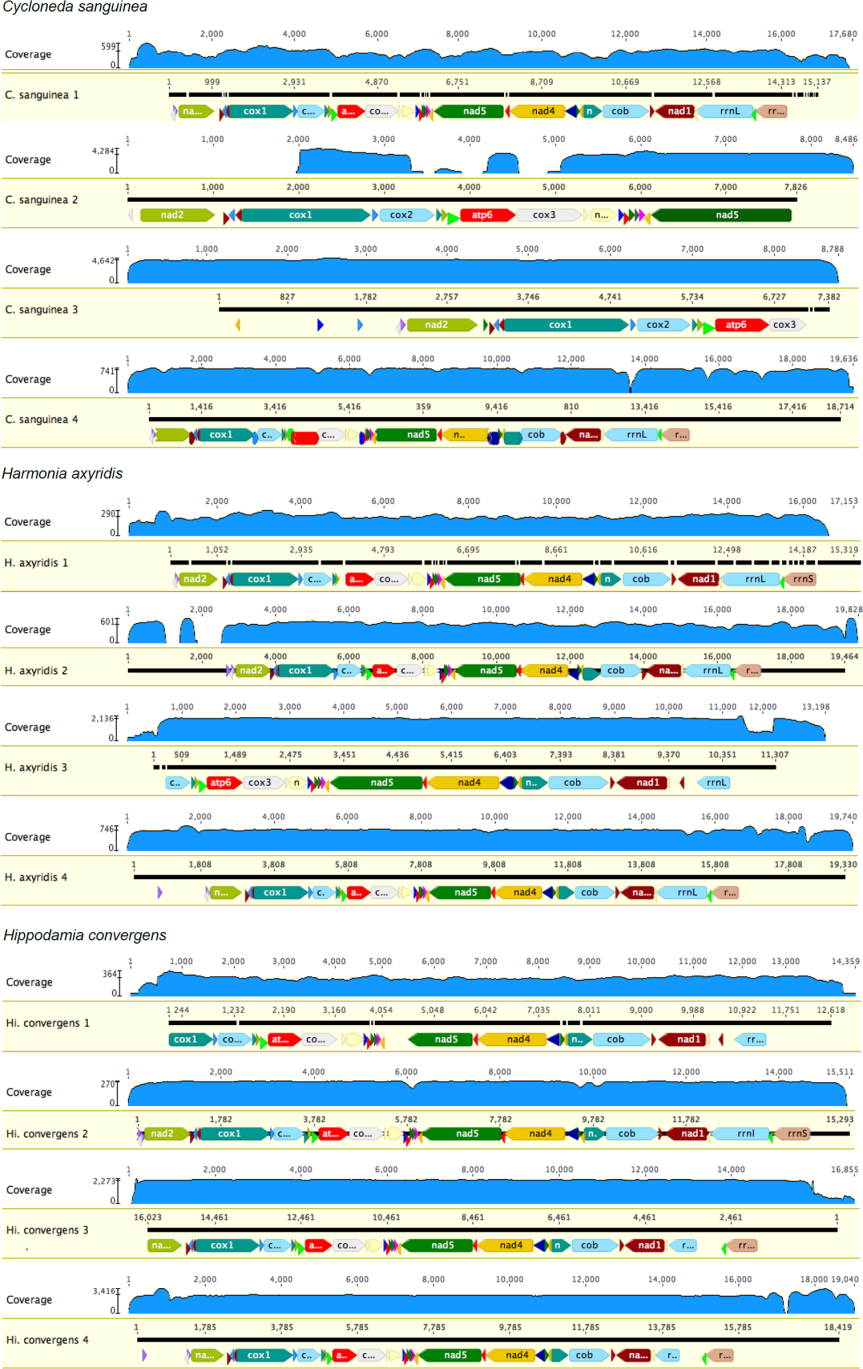
Coverage of the mitogenomes obtained for each species using the four methods. The minimum and maximum (presented in log-scale) is indicated on the ordinate of each figure and the coverage for each nucleotide position is indicated by the height of the blue line. The black bar represents the consensus nucleotide sequence and horizontal thin lines represent gaps. In each figure, the number after the species mitogenome name indicates the method used to assemble and annotate.

The number of raw reads after quality control for the pooled library was 1,663,759 and for the single libraries: *C*. *sanguinea* 4,587,115; *H*. *axyridis* 3,233,855; *Hi*. *convergens* 2,827,085. The lower number of raw reads in the pooled library might have been related to pooling of the species, the longer insert size (850 versus 550 bp) and the use of the MiSeq platform. There was variation in the number of reads mapped to the assembled mitogenomes across pipelines (Table 2), which was directly related to the coverage (Fig 2), but not related to the number of raw reads. Different methods conferred different average coverage. The mitogenomes of *C*. *sanguinea* and *Hi*. *convergens* were elucidated for the first time.

**Table 2:** Coccinellid mitogenomes obtained using different methods.

	*Cycloneda sanguinea*	*Harmonia axyridis*	*Hippodamia convergens*
**Method 1 (Pooled_*de novo*_customized annotation)**			
Length (bp)	15,137	15,319	12,618
Protein-coding genes (PCGs)	13	13	12
tRNAs	22	19	15
rRNAs	2	2	1
Number of reads mapped	4,884	4,857	3,807
Mean coverage and std dev	71.8±68.3	74.3±44.2	68.2±39.8
GenBank	KJ778883	KJ778886	KJ778888
**Method 2 (Pooled_MITObim_MITOS)**			
Length (bp)	7,826	19,464	15,293
PCGs	9	13	13
tRNAs	14	22	22
rRNAs	0	2	2
Number of reads mapped	16,797	12,030	12,652
Mean coverage and std dev	489.5±679.9	134.0±86.2	200.4±46.0
GenBank	KU877027	KU877028	KU877030
**Method 3 (Single_*de novo*_customized annotation)**			
Length (bp)	7,382	11,307	16,023
PCGs	7	12	13
tRNAs	11	14	19
rRNAs	0	1	2
Number of reads mapped	87,672	47,512	94,994
Mean coverage and std dev	2,266.7±495.4	816.8±329.1	1,239.9±366.3
GenBank	KX755335	KX755334	KX755332
**Method 4 (Single_MITObim_MITOS)**			
Length (bp)	18,715	19,330	18,419
PCGs	13	13	13
tRNAs	22	22	22
rRNAs	2	2	2
Number of reads mapped	38,213	20,022	36,543
Mean coverage and std dev	474.7±161.19	222.2±83.8	411.5±404.4
GenBank	KU877170	KX755330	KX755331

### Mitogenome completeness

The mitogenomes of the three ladybird beetle species differed across pipelines within species regarding completeness (number of genes) (Table 2). Overall, the coverage was good (>10 [35]) except for parts of *C*. *sanguinea* method 2 and a few nucleotides in the other mitogenomes (Fig 2). The mitogenomes assembled *de novo* with customized annotation generally were incomplete, lacking some PCGs and/or tRNAs. Overall for all the species, assembly using MITObim (methods 2 and 4) resulted in the complete number of mito-genes, whether species were pooled or not in the libraries. The only exception was for the *C*. *sanguinea* mitogenome obtained by method 2, which for unknown reasons had no or low coverage in several regions (Fig 2). However, even for methods 2 and 4, it is not clear if the length is complete, as none of the sequences could be circularized.

### Mitogenome sequence identities across pipelines

**Fig 3:**
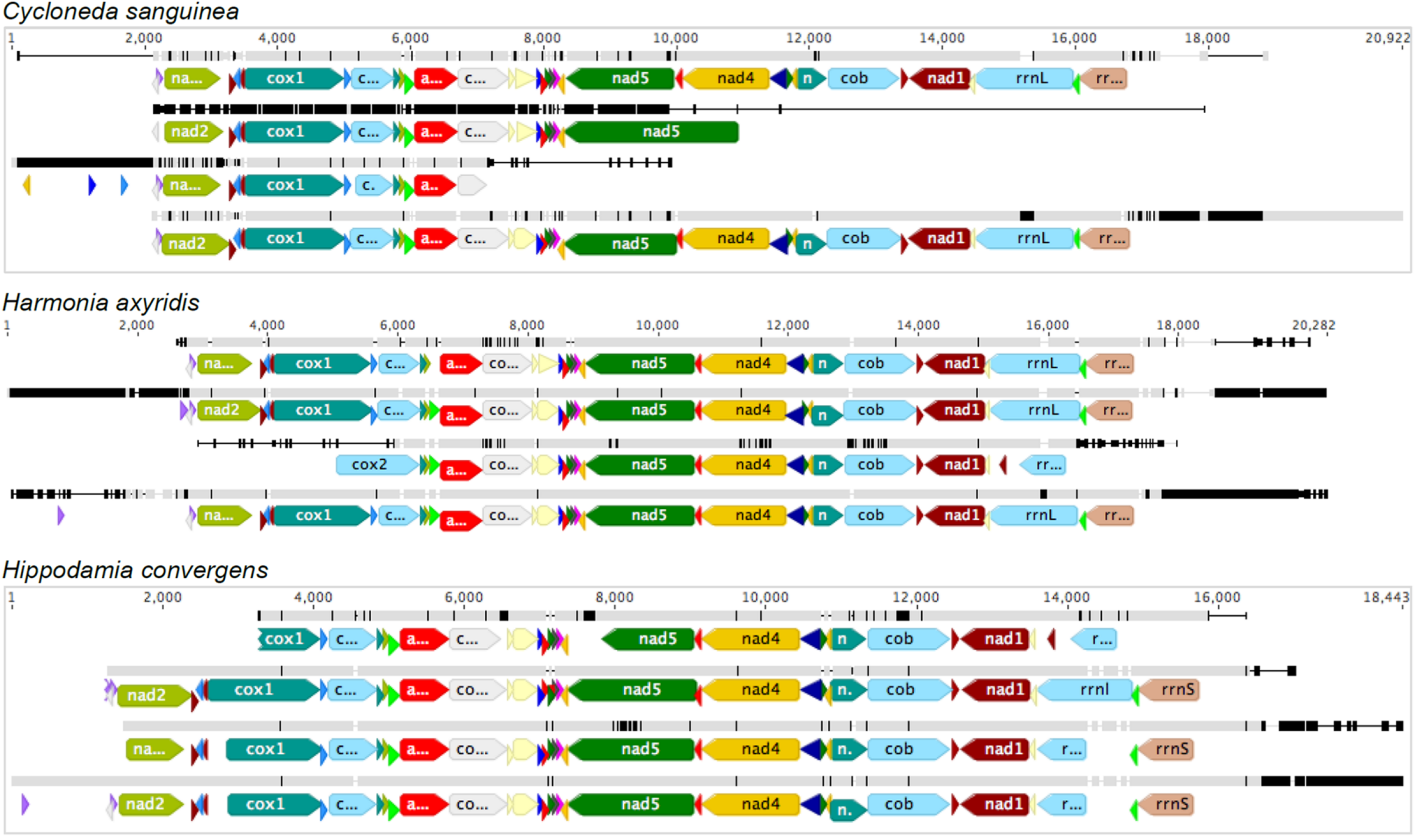
Alignment and comparison of the annotations and similarity of the mitogenomes of *C*. *sanguinea*, *H*. *axyridis* and *Hi*. *convergens* using the four methods. The annotated mitogenomes are in color, with arrows representing the transcriptional sense of the gene, and small arrows representing tRNA genes. Grey bars represent similar nucleotides and vertical black bars represent dissimilar nucleotides in the target sequence compared to the consensus sequence, and horizontal black lines represent gaps in the target sequence. The four methods are displayed in order from the top for each species.

In addition to differences in completeness, there were differences in the sequence identities across pipelines for a same species. Table 3 presents the % identity for all pairwise comparisons of the entire mitogenomes obtained by the four methods for all species, and Fig 3 presents the regions of dissimilarity and gaps among the four methods for each species. Comparing methods 1 vs 2 and 3 vs 4 would reveal the effect of the assembly and annotation methods; comparing methods 1 vs 3 and 2 vs 4 would reveal the effect of the sequencing method (species pooling, library insert size and sequencing platform); and comparing methods 1 vs 4 and 2 vs 3 would reveal the combined interaction effects of the two sets of factors assembly and annotation method with sequencing method. The low identities (Table 3) found among the mitogenome sequences are related in part to gaps in one of the assemblies, but there are substantial differences in the sequences even in regions without gaps (Fig 3).

**Table 3:** Identity (%) among the mitogenomes generated by the different methods.

Mitogenomes	Identity (%)					
	Assembly and annotation method		# species pooling		Interaction of assembly, annotation and species pooling	
	1 vs 2	3 vs 4	1 vs 3	2 vs 4	1 vs 4	2 vs 3
*Cycloneda sanguinea*	40.17	66.55	52.87	38.19	90.10	51.57
*Harmonia axyridis*	91.06	72.85	73.93	83.41	87.38	74.06
*Hippodamia convergens*	93.60	41.15	43.70	96.71	93.51	44.37

The effect of the assembly and annotation method differed depending on the species. The mitogenome sequences of a species from the pooled library were more similar (1 vs 2) than from the separate species libraries (3 vs 4), suggesting that the different assembly and annotation methods generated greater differences in the sequences when the species were in individual libraries than when pooled in the same library. However, mitogenomes from *C*. *sanguinea* showed the opposite; being more similar from the individual species library.

Similarly, the effect of sequencing method differed depending on the assembly and annotation method and the species. For two of the species, the mitogenome sequences of a species were more similar when assembled by MITObim (2 vs 4) that when done *de novo* (1 vs 3). This indicated that the effect of sequencing method was greater when using *de novo* assembly than MITObim. Again, mitogenomes from *C*. *sanguinea* showed the opposite; being more similar using *de novo* assembly than MITObim.

Finally, for all species, mitogenome sequences of a species were more similar comparing pooled *de novo* assembly with single-species MITObim assembly (1 vs 4) than methods 2 vs 3. Thus, the effects of the two sets of factors depended on each other.

The gray and black bars above the annotated and aligned sequences in Fig 3 show the points of dissimilarity among the four methods for each species mitogenomes. While some of the dissimilarity may have been due to low coverage (*e*.*g*., *C*. *sanguinea* method 2 for *nad*2 through the first half of *cox*1), dissimiliarity persisted even when coverage was high (*e*.*g*., *C*. *sanguinea* method 2, second half of *cox*1 to the first half of *cox*2 and the second half of *cox*3 to the first half of *nad*5). Overall, there was no clear relation between low coverage and dissimilarity among the sequences.

**Fig 4:**
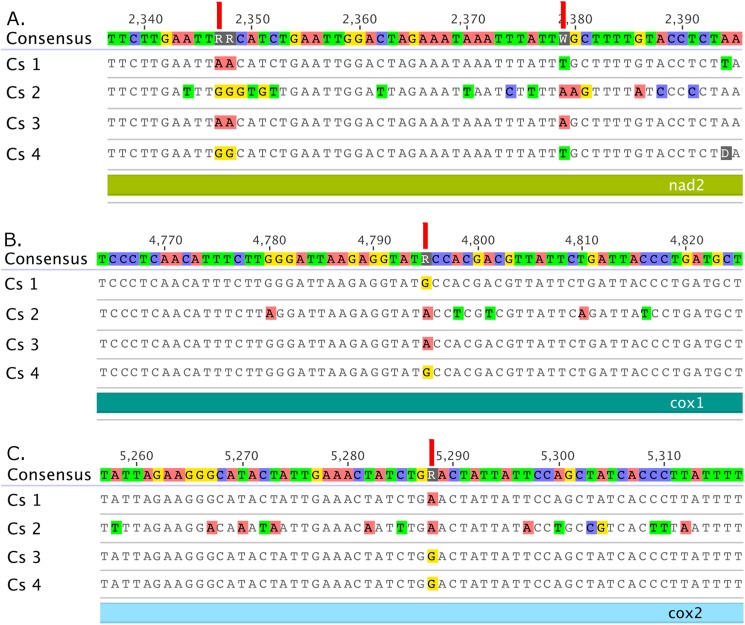
Examples of dissimilarities observed in the mitogenomes of *C*. *sanguinea* (Cs) generated by different pipelines. The alignments are parts of (A) *nad*2, (B) *cox*1 and (C) *cox*2 genes, with the consensus sequence on the top and the four methods below (Cs1, Cs2, Cs3, and Cs4).

A more detailed examination of the differences generated by the four pipelines is shown in Fig 4 for *C*. *sanguinea*. For example, at position 2,347 of the consensus sequence of the *nad*2 gene, methods 1 and 3 resulted in an adenine (A), but methods 2 and 4 resulted in a guanine (G), indicating that different assembly methods gave different sequences. At position 5,288, methods 1 and 2 had an A and methods 3 and 4 had a G, indicating that different sequencing methods gave different sequences. Finally at positions 2,379 and 4,795, methods 1 and 4 resulted in the same base (thymine (T) or G) while methods 2 and 3 resulted in a different base (A), indicating that both sequencing and assembly methods matter. These differences were not associated with insufficient (considered to be <10-fold redundancy [35]) or differences in coverage (Table 4).

**Table 4:** Coverage for *C*. *sanguinea* mitogenomes at the indicated consensus positions for the four methods.

Gene	*nad*2	*nad*2	*cox*1	*cox*1	*cox*2
Consensus position (bp)	2,347	2,379	3,796	4,795	5,288
Method 1	45	41	72	122	90
Method 2	1	1	1	1,039	433
Method 3	2,324	2,282	1,924	2,290	2,515
Method 4	495	423	518	600	563

### Mitogenome annotations

As expected, the annotations of a species varied across different combinations of libraries and assembly/annotation methods, even when the same annotation method was used (Figs 2 and 3). Methods 1 and 4 provided the most similar annotations for *C*. *sanguinea*, going from the gene *trn*Q to *rrn*S, although with a length difference in the *nad*2 gene. These were the only methods providing a sequence with all of the expected mito-genes. Methods 2 and 3 provided shorter mitogenomes with fewer annotated genes. For *H*. *axyridis*, methods 2, and 4 provided sequences with all of the expected mito-genes. These were equivalent to that from method 1, except for the length of the *nad*2 gene, which in method 1 was split into two pieces, and in the presence and position of *trn*I. Method 3 provided the most incomplete annotation (missing initial tRNAs and *cox*1, and *rrn*S at the other end) and had different gene size annotations for *cox*2, *rrn*L, *rrn*S, although it was the only one that presented *trn*W before *rrn*L gene. The gene *nad*2 is also smaller and presented immediately after *rrn*L. For *Hi*. *convergens*, methods 2 and 4 provided sequences with all of the expected mito-genes, starting from *trn*I to *rrn*S. The genes *nad*2, *cox*1 and *rrn*L differed in size according to the pipeline used. Method 1 provided a shorter mitogenome sequence and an incomplete annotation, although it was the only one that presented *trn*W before *rrn*L gene. Overall, method 4 (use of MITObim to assemble and MITOS to annotate) seemed to be the one that provided the most complete annotation for all the species.

## Discussion

Our main result is that for three related species, different sequencing and assembly/annotation methods produced dissimilar mitogenome sequences within each of the three species. In many cases, different library data sets resulted in different nucleotides at the same position when the assembler was the same; the same library data set resulted in different nucleotides when the assembler was different; and different library data sets and assemblers resulted in different nucleotides. In addition, the effects of this methodological variation differed among the species.

This sensitive dependence on sequencing and assembly/annotation method raises concerns. First, if we had relied on only one method, we would not know to question the veracity of the resulting mitogenome sequence. Three of the four methods produced at least one complete mitogenome sequence for the three species, and the remaining method might also produce complete sequences if we had used it on more species. Second, good coverage (>10-fold redundancy) did not eliminate dissimilarity, but might provide illusory confidence in the accuracy of the resulting sequence. Third, the methodological effects differed among the three species, suggesting that finding a “best” method may be challenging, possibly because the mitogenomes of some species may be more difficult to elucidate than others. Although MITObim often provided the mitogenomes with the full mito-genes (Table 2), it did not always do so (*e*.*g*., *C*. *sanguinea* with the pooled library).

**Fig 5:**
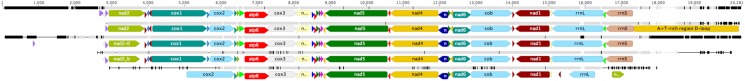
Comparison of the mitogenome sequences of *H*. *axyridis* obtained in this work with *H*. *axyridis* (KR108208) [38] using standard parameters in MAFFT v7.017 [34]. The KR108208 has 16,387 bp, 13 PCGs, 20 tRNAs (missing *trn*I and *trn*Q) and 2 rRNAs. The mitogenomes sequenced here are displayed with highest similarity on top. From the top to the bottom are: the mitogenome obtained by method 2; KR108208; followed by methods 4, 1 and 3, respectively. The annotated mitogenomes are in color, with arrows representing the transcriptional sense of the gene, and small arrows representing tRNA genes. Grey bars represent similar nucleotides and vertical black bars represent dissimilar nucleotides in the target sequence compared to the consensus sequence, and horizontal black lines represent gaps in the target sequence.

We expected that the individual species libraries on the HiSeq platform with smaller insert size would provide higher mitogenome completeness because of the greater sequencing depth and elimination of complications due to the relatedness of the species. However, the mitogenomes of all three species obtained by method 3 (single libraries and *de novo* assembly) were incomplete, despite the high coverage and sequencing depth. Pooling different species in a same library without tagging did not seem to affect significantly the quality of the resulting sequences. Other researchers have successfully pooled species [22,36,37]. According to Tang et al. [22] species as close as congeners can be pooled and mitogenomes successfully assembled with more than 50 species in a single lane of HiSeq 2000 (with ≤100 ng genomic DNA). Although the creation of chimeras is a concern, especially among closely related species, in our case, their effect was minimal as the annotations were equivalent across sequencing pipelines (Figs 2 and 3), with no differences in gene order or transcriptional direction, and only a few differences in gene length. We compared the identity of the mitogenome sequences of *H*. *axyridis* obtained in this work with the *H*. *axyridis* KR108208 mitogenome deposited in GenBank [38]. The KR108208 sequence is likely to be a high quality sequence, although it is missing *trn*I and *trn*Q, because it was elucidated using LR-PCR and primer walking coupled with Sanger sequencing, and BLAST search in GenBank and tRNAscan-SE to assemble and annotate the mitogenome (personal communication, SJ Wei). There was 91.07, 97.81, 74.05 and 89.62% identity, respectively with our four sequences (Fig 5). The dissimilarities mostly occurred in non-coding regions such as the A+T-rich region and in the terminus. Method 2 was most similar to this comparator and perhaps provided better results for *H*. *axyridis* mitogenome elucidation and annotation. However, method 4 differed mainly in the A+T-rich region that was missing from the others.

Our results led us to question the reliability of the mitogenome sequences deposited in GenBank. In general, these sequences do not have information about the sequencing, assembly or annotation method, so it is not possible to evaluate independently the completeness or accuracy of the deposited sequences. We examined the 2,469 complete Insecta mitogenomes deposited at GenBank and found 60 species with mitogenomes provided by different research groups on species that did not have subspecies or biotypes. Of those, eight species had mitogenome sequence identity <90%. For example, *Bemisia afer*, KR819174 (15,300 bp) and NC_024056 (14,968 bp), had only with 67.94% similarity. The other seven species are: *Apis florea*; *Choaspes benjaminii*; *Eurema hecabe*; *Gastrimargus marmoratus*; *Luehdorfia chinensis*; *Papilio helenus*; and *Rondotia menciana*. In addition, Lv et al. [39] found mitogenomes of *Nilaparvata lugens* (14,364–14,371 bp) that were much smaller than that reported previously (15,190 bp) [40]. Unfortunately, in many cases, the methods used to elucidate and annotate the mitogenomes were not provided in the GenBank deposition or in the associated publication (when available). While it is possible that these dissimilarities might be due to misidentification of the species, several of the eight species are easy to identify, so this is an unlikely explanation for all of the large intraspecific variations in sequence similarity in GenBank. Studies on intraspecific variation in parts of the mitogenome typically reported identities >97% [8,39,41]. Hence, similarities below 90% are much lower than expected for intraspecific variation, and are more characteristic of interspecific or intergeneric variation.

We suggest that there is a need to adopt additional reporting requirements and a validation method for mitogenome deposition at public DNA databases. Based on our results, at minimum, information about the sequencing, assembly and annotation methods are relevant, and should be associated with a Sequence Read Archive (SRA) deposition. In addition, coverage should be reported, although this should not be relied on to validate the sequence. As biological inferences in several biological fields have been made and are being made relying on these deposited sequences, a check on accuracy is an issue that urgently needs to be addressed by the scientific community as more and more sequences are expected to be provided (Fig 1) through different bioinformatics pipelines [42].

More broadly, we suggest that guidelines need to be developed by the scientific community to verify the accuracy of mitogenome sequences prior to deposition, perhaps modeled on those for qPCR analysis (The MIQE guidelines) [43]. Until then, one alternative to verify accuracy before database deposition might be based on an approach similar to that used by Tang et al. [22] to assure sequence quality when 49 species were pooled in a library. This alternative could include the use of more than one assembly method (a hybrid combination of *de novo* and reference genome assemblers might be an interesting choice), concatenate their scaffolds in another assembler, and map the reads to identify regions with lower coverage. Sanger sequencing could be used to verify regions that are assembled only by one of the assemblers with lower coverage or that exhibit dissimilarity or gaps among assemblers.

## Conclusions

Mitogenome completeness and sequence accuracy was affected by a number of methodological factors in the experimental design. The scientific community urgently needs to address the issue related to the lack of information regarding the quality of the mitogenomes sequences deposited considering that they have been generated by different methods for sequencing, assembly and annotation, among other factors. The deposition rate of mitogenomes has exploded due to the technical advancements in DNA sequencing methods and bioinformatics pipelines. Guidelines for verifying and validating mitogenome sequences should be discussed and established to assure mitogenome quality for future studies.
